# Do mothers accurately identify their child’s overweight/obesity status during early childhood? Evidence from a nationally representative cohort study

**DOI:** 10.1186/s12966-018-0688-y

**Published:** 2018-06-19

**Authors:** Michelle Queally, Edel Doherty, Karen Matvienko-Sikar, Elaine Toomey, John Cullinan, Janas M. Harrington, Patricia M. Kearney, on behalf of the Choosing Healthy Eating for Infant Health (CHErIsH) study team

**Affiliations:** 1 Discipline of Economics, JE Cairnes School of Business and Economics, National University of Ireland, Galway, Ireland; 2 Health Behaviour Change Research Group, School of Psychology, National University of Ireland Galway, Galway, Ireland; 3 School of Public Health, University College Cork, Cork, Ireland

**Keywords:** Childhood obesity, Maternal perception, Preschool overweight obesity identification

## Abstract

**Background:**

Maternal recognition of overweight/obesity during early childhood is a key determinant in achieving healthy weight status in children. The aim of this study is to 1) investigate maternal perceptions of their child weight, focusing on whether or not mothers accurately identify if their child is overweight or obese at three years old and five years old; 2) identify the factors influencing maternal misperceptions regarding their child’s weight at three years old and five years old, 3) ascertain if a failure to recognize overweight/obesity at three years old is associated with the likelihood of doing so at five years old.

**Methods:**

Using two waves of the longitudinal Growing Up in Ireland study data regarding child, maternal, and household characteristics as well as healthcare access and utilization variables were obtained for mothers when their children are three and five years old respectively. Multivariate logistic analysis was used to examine the factors associated with mothers inaccurately perceiving their child to be of normal weight status when the child is in fact either clinically overweight or obese.

**Results:**

In wave 2, 22% of mothers failed to accurately identify their child to be overweight or obese. This inaccuracy decreased to 18% in wave 3. A failure of mothers to identify their child’s overweight/obesity was more likely to occur if the child was a girl (OR: 1.25) (OR: 1.37), had a higher birth weight (OR:1.00), if the mother was obese (OR: 1.50), (OR: 1.72) or working (OR:1.25) (OR:1.16) in wave 2 and wave 3, respectively. Other factors affecting the odds of misperceiving child’s weight include gestation age, income and urban living.

**Conclusion:**

These findings suggest that mothers of overweight or obese three and five year olds show poor awareness of their child’s weight status. Both child and mother characteristics play a role in influencing this awareness. Mothers unable to accurately identify their child’s overweight or obesity status at three years old are likely to do again when the child is five years old. This study highlights the need for increased support to help improve mothers’ understanding of healthy body size in preschool aged children.

## Background

Despite the global prevalence of obesity a high proportion of parents continue to misclassify their child’s weight status [35, 40]. The majority of studies demonstrate parental underestimation of child weight status [12, 16, 32]. Considerable research has been devoted to parental perceptions of children’s weight during late childhood or adolescent stages [1, 2, 36] and also during the toddler years [5, 14, 19, 26]. Although the literature suggests that parental awareness of weight problems may be particularly low for younger children [15, 29, 35], there has been relatively less attention paid to examining parental weight perception during the younger years particularly for preschool children [7, 16, 24]. Moreover, the factors that influence parental perceptions of child weight during preschool age are less documented to that of when the child is older.

Maternal ability to correctly identify their child's weight status during the preschool years is important. The initial step in addressing childhood obesity is the recognition of the problem [44]. If mothers are unable or unwilling to recognize that their child is overweight, they cannot intervene early to prevent further excess weight gain [41]. Potentially important factors that may influence maternal weight perceptions during the preschool years have not yet been examined in the literature. For example child gestation age, whether or not the child has siblings, whether or not the child was breastfed, self-reported health status and healthcare access and engagement. Examining if healthcare professionals play a role in influencing maternal perceptions of child weight is important in terms of informing childhood obesity prevention interventions during the early years. Additionally, no study has examined if mothers become better able to correctly perceive their child’s weight status within the preschool years. Ascertaining if failing to accurately identify children’s weight at toddler age influences the likelihood of doing so at preschool age, along with whether or not the factors influencing perception change over time would help inform more timely interventions related to preventing childhood obesity. In addition to exploring the role of these variables above, it would be informative to examine whether factors that were found to be associated with misperceptions of older children’s weight status in previous studies were significant for preschool children also. These factors include, maternal obesity and maternal education [12] and childbirth weight [9]. Correlations between child weight misperceptions and socio-economic status, race/ethnicity and gender are mixed [7, 29, 37].

The aims of this study are to 1) investigate maternal perceptions of their child weight, focusing on whether or not mothers accurately identify if their child is overweight or obese at three years old and five years old; 2) identify the factors influencing maternal misperceptions regarding their child’s weight at three years old and five years old, 3) ascertain if a failure to recognize overweight/obesity at three years old is associated with the likelihood of doing so at five years old.

## Methods

### GUI data

Data for this analysis derives from the Growing Up in Ireland (GUI) study^1^. The GUI is a nationally representative face-to-face survey of children living in Ireland, and aims to inform policy in relation to children, young people and families [18]. The GUI study commenced in 2006 and follows the progress of two groups of children: 8,568 9-year-olds (Child Cohort) and 11,134 9-month-olds (Infant Cohort) [43]. For this analysis data were used from the Infant Cohort. The Infant Cohort follow-up waves were completed when the child was aged three years, five years and seven/eight years (postal). This study used data from when the children were three years old and five years old respectively. Wave 2 when the children were three years old collected information from 9793 families and wave 3 when the children were five years old contained information from 9001 families^2^.

In both waves, the primary caregiver was asked: Which of these best describes your child’s weight? In wave 2 [30], the response is grouped as follows: (1) Underweight; (2) Normal weight; (3) Somewhat overweight; (4) Refusal; and (5) Don’t know. In wave 3, [39] the response is grouped slightly different as follows: (1) Underweight; (2) Normal weight; (3) Somewhat overweight\ Very Overweight; and (4) Don’t know. In addition to these variables, anthropometric measurements were taken according to standard procedures for children at both waves. Child height was measured by trained research staff during the home interview using a Leicester portable height stick. Child weight was measured using SECA digital scales.

### Description of variables

A literature review was undertaken in order to guide the selection of variables included in the statistical analysis. For the analysis, data relating to child and mother characteristics, healthcare access and utilisation variables and household characteristics were extracted. For the child, data extracted included gender, child birth weight, gestation age, whether or not the child was breastfed, whether the child had siblings and child health status as reported by the mother. For the mother, data extracted included education, obesity status, age, self-reported health, employment status, whether or not the mother was born in Ireland and marital status. Healthcare access and utilisation variables for the child included medical card status, private health insurance, the number of doctor visits, paediatric visits, public health nurse visits, practice nurse visits and the number of nights spent in hospital by the child. Whether or not a child had a medical card and or private health insurance is important in an Irish context wherein medical cards are allocated to individuals based largely on economic deprivation. Medical cards allow free access to general practitioner (GP) services at point of care. Private health insurance is often used to gain faster access to hospital services but incurs a cost(s). Household characteristics include income, as well as whether the household was located in a rural or urban area. It is worth noting that missing data on several of the variables reduced the sample sizes somewhat for analysis^3^

### Empirical approach

For this analysis, in order to ensure the child’s clinical body mass index (BMI) measure corresponded to the categories of the mothers’ perception of their child’s weight, the child’s clinical BMI was aggregated into similar groups to that of the mother’s perceived weight [10] of her child. The defined groups were (1) healthy weight and (2) overweight and obese. In order to generate these groups, BMI was calculated using the children’s weight and height centiles based on 1990 UK reference curves [11]. The BMI categories were then generated using the International Obesity Task Force cutoffs [10]. Underweight children were excluded from the analysis^4^. Mothers who inaccurately identified their child to be overweight/obese when the child was within a healthy weight status was also examined. These were excluded from analysis due to small numbers^5^. In order to investigate evidence of any inaccuracies regarding identification of overweight/obesity, the clinical BMI measure of each child in the sample was compared to the mother’s perception of their child’s weight status^6^.

In total, three multivariate binary logistic models were estimated to explore factors associated with maternal misclassification. The procedure used by Cullinan and Crawley (2017) to derive the maternal weight perceptions was adopted. One type of binary misclassification variable for wave 2 and wave 3 named ‘*MisclassifyW2*’and ‘*MisclassifyW3*’ respectively, was calculated. In model one “*MisclassifyW2*” is the dependent variable, which describes if the mother perceived the child to be of normal weight when the child was [clinically] overweight/obese at three years old. Model two “*MisclassifyW3*” is the dependent variable, which describes if the mother perceived the child to be of normal weight when the child was [clinically] overweight/obese at five years old. In the third model “*MisPredict*” is the dependent variable, which describes whether mothers’ who misclassified their child’s weight at three years old is a predictor of misclassifying their child at five years old. In the final models, a full set of covariates are included, as described in Table 1, as explanatory variables. Therefore for each wave, a model is estimated which includes all the covariates simultaneously. The models apply weights to ensure that the data is nationally representative.

**Table 1:** Descriptive Statistics of the Sample and Description of the Variables used

Variable Name	Variable Description	Wave 2 at 3 years old % or Mean (SD)	Wave 3 at 5 years old % or Mean (SD)
Child characteristics			
*Girl*	= 1 if child is female; = 0 if male	49.93%	49.87%
*Birth weight*	Child’s birth weight in grams	3514 (523)	3514 (525)
*Gestation age*	Child’s gestation age in weeks	39.57 (1.94)	39.53 (2.01)
*Breast fed*	= 1 if child was breastfed as a baby; =0 if otherwise	60.45%	61.59%
*Siblings*	= 1 if child has siblings; = 0 if otherwise	80.41%	81.66%
*Child good health (as self- reported by the mother)*	= 1 if mother states her child’s health is excellent, very good or good; =0 if otherwise	98.19%	98.52%
*Mother characteristics*			
*Education*	= Primary/ lower secondary education (reference level)	8.05%	6.28%
	= Complete secondary	13.77%	11.27%
	= Technical/vocational/non-degree qualification	36.86%	42.08%
	= Degree/postgraduate	41.32%	40.38%
*Obesity status*	= 1 if mother is obese (BMI >= 30); = 0 otherwise	17.32%	19.18%
*Age*	Age of mother (this is the age of the mother in continuous years when the baby was 9 months old; the subsequent waves of GUI data only contained categories for mothers’ age)	31.84 (5.16)	32.13 (5.04)
*Mother good health (as self- reported by the mother)*	=1 if mother states her health is excellent, very good or good; =0 if otherwise	93.71%	95.54%
*Married*	= 1 if mother is married or cohabiting; =0 if otherwise	72.87%	75.69%
*Working*	= 1 if mother is working; = 0 if otherwise	57.44%	58.21%
*Irish*	= 1 if mother is Irish; = 0 if otherwise	82.59%	84.34%
*Healthcare access and utilisation variables*			
*Medical card*	= 1 if child is covered by a medical card; 0 if otherwise	35.60%	39.71%
*Private health insurance*	= 1 if child is covered by private health insurance; 0 if otherwise	54.13%	50.68%
*Doctor visits*	= number of doctor visits by child in the past year	2.43 (2.78)	1.77 (1.94)
*Pediatric visits*	= number of visits by child to pediatrician or hospital consultant in the past year	0.499 (1.16)	0.426 (0.952)
*Public health nurse visits*	= number of public health nurse visits by child in the past year	0.355 (0.647)	0.139 (0.424)
*Practice nurse visits*	= number of practice nurse visits by child in the past year	0.129 (0.519)	0.108 (0.382)
*Hospital nights*	= number of nights spent in hospital by child since previous wave of data collection	0.633 (2.45)	0.277 (0.966)
*Hous ehold characteristics*			
*Equivalised income*^*a*^	Equivalised household income in quintiles		
	Lowest (base)	19.01%	17.45%
	2nd	18.32%	17.94%
	3rd	19.81%	19.88%
	4th	20.35%	21.35%
	Highest	22.51%	23.38%
*Urban*	= 1 if household in urban area; =0 otherwise	42.72%	38.55%
Observations		7587	6417

^a^The equivalised disposable income is the total income of a household, after tax and other deductions, that is available for spending or saving, divided by the number of household members converted into equalised adults; household members are equalised or made equivalent by weighting each according to their age, using the so-called modified OECD equivalence scale

## Results

Table 1 provides an overview of the variables used in the models and the number of observations in both waves.

In particular, table 1 provides details of the child, maternal, household and healthcare utilisation characteristics. Across each wave almost half of the children were females and the average birth weight was 3514 grams. There was a slight increase in the percentage of mothers who reported that their child was breastfed in wave 2 from 60% to 62% in wave 3. The percentage of children who had siblings increased from 80% in wave 2 to 82% in wave 3. Over 98% of mothers rated their child to be in good health across both waves. Maternal characteristics indicated that over 40% of mothers in the sample had attained third level education in each wave. There was a 2% increase in the percentage of mothers who were obese in wave 3, from 17% to 19%. There was a 3% increase in mothers who were married from 73% in wave 2 to 76% in wave 3. There was a 1% increase in mothers who reported themselves as working from 57% in wave 2 to 58% in wave 3. The majority of mothers described themselves as Irish born (83%). There was a 3% decrease in the percentage of mothers who reported having private health insurance from 54% in wave 2 to 51% in wave 3. There was a 4% increase in mothers who have medical cards from 36% in wave 2 to 40% in wave 3. In wave 2 and wave 3, 23% of households are reported to be in the higher income decile. There was a 4% decrease of mothers reported as living in an urban area from 43% in wave 2 to 39% in wave 3.

Table 2 indicates the percentage of children within the sample used for this analysis who are normal weight, overweight and obese as clinically defined by measured BMI, across each wave. This table also describes the percentage of mothers who perceived their child’s weight status to be normal weight and overweight and obese within this sample across each wave.

**Table 2:** Child Clinical Weight Classification and Mothers Perceived Weight Classification

	Clinical Weight Measure	Maternal Classification
	Wave 2 (three years old)	
Normal weight	74.34 % (5,640)	96.74% (7,340)
Overweight & Obese	25.66% (1,947)	3.26 % (247)
Total	7,587	7,587
	Wave 3 (five years old)	
Normal weight	77.54% ( 4,976)	95.54 % (6,131)
Overweight & Obese	22.46 % (1,441)	4.46% (286)
Total	6,417	6,417

Table 2 shows that just over 22% of mothers failed to accurately identify their child to be overweight or obese in wave 2. This percentage of mothers who failed to accurately identify their child as being overweight or obese decreased by 4% to 18% in wave 3. Almost three quarters (74%) (N=5,640) of children in this study were within a normal weight status, as indicated by their measured BMI at three years old. The percentage of children within a normal weight status as clinically measured increased to 75% (N=4,976) in wave 3. There was a 3.2% decrease in the number of children who were clinically defined as overweight or obese from 25.66% in wave 2 to 22.45% in wave 3.

Table 3 presents the odds ratio from the multivariate logistic regression analyses which examined covariates associated with mothers’ misclassifying their child’s weight status. Model 1 and 2 show the findings from the logistic models estimated for children when they are three years old and five years old respectively. The third model includes an explanatory variable that describes if a mother misclassified their child’s weight status when the child was three years old.

**Table 3:** Multivariate Logistic Odds Ratio Models of Mother’s Misclassifications of Child Overweight Status

	Mode1 1: MisclassifyW2 (dependent variable =1 if mother misclassified the child’s weight status at three years old; 0 otherwise) (95% Confidence Intervals)	Model 2: MisclassifyW3 (dependent variable =1 if mother misclassified the child’s weight status at five years old; 0 otherwise) (95% Confidence Intervals)	Model 3: MisPredict (same as model 2 with the dependent variable from model 1 added as an additional explanatory variable) (95% Confidence Intervals)
Misclassify			7.261^a^ (6.13-8.59)
* Girl*	1.247^a^ (1.09-1.42)	1.366^a^ (1.17- 1. 59)	1.384^a^ (1. 17- 1.63)
* Birth weight*	1.001^a^ (1.00-1.00)	1.001^a^ (1.00- 1.00)	1.000^a^ (1.00-1.00)
* Gestation age*	0.926^a^ (0.88-0.96)	0.967 (0.92-1.01)	0.998 (0.94-1.05)
* Breast fed*	0.959 (0.83-1.11)	0.917 (0.77-1.08)	0.894 (0.74- 1.07)
* Siblings*	0.853 (0.71-1.01)	0.855 (0.66-1.09)	0.953 (0.71-1.26)
* Child good health*	1.390 (0.72-2.43)	0.612 (0.32-1.14)	0.775 (0.38- 1.58)
* Education* Complete Secondary	0.864 (0.65- 1.13)	0.830 (0.58-1.17)	0.817 (0.53-1.20)
* Technical/vocational/non-degree*	0.882 (0.68- 1.13)	0.816 (0.59- 1.11)	0.836 (0.59-1.18)
* Degree/postgraduate*	0.850 (0.64- 1.11)	0.702^b^ (0.49- .99)	0.675^b^ (0.46-.97)
* Obese*	1.508^a^ (1.28- 1.77)	1.721^a^ (1.43- 2.06)	1.630^a^ (1.32-2.00)
* Age*	3.126 (0.34- 26.6)	2.142 (0.22- 20.51)	2.166 (0.16-27.86)
* Mother good health*	1.078 (0.81- 1.42)	1.312 0.87- 1.96)	1.233 (0.80- 1.89)
* Married*	0.963 (0.80-1.14)	0.883 (0.71- 1.08)	0.924 (0.73- 1.16)
* Working*	1.254^a^ (1.07-1.45)	1.168^c^ (0.98-1.38)	1.125 (0.92-1.36)
* Irish*	1.175 (0.96- 1.43)	0.818^c^ (0.64- 1.03)	0.719^a^ (0.55-.92)
* Medical card*	1.024 (0.84- 1.23)	1.141 (0.92-1.40)	1.161 (0.91-1.47)
* Private health insurance*	0.776^a^ (0.65-0.92)	0.982 (0.80-1.19)	1.000 (0.80-1.23)
* Doctor visits*	0.993 (0.96- 1.02)	1.000 (0.95-1.04)	1.006 (0.95-1.05)
* Pediatric visits*	1.061 (1.01-1.14)	0.982 (0.90- 1.06)	0.946 (0.85- 1.04)
* Public health nurse visits*	1.079 (0.97-1.19)	0.963 (0.84- 1.09)	0.938 (0.81-1.08)
* Practice nurse visits*	0.896^c^ (0.78-1.02)	1.044 (0.84-1.28)	1.126 (0.89-1.41)
* Hospital nights*	1.010 (0.74`- 1.07)	0.984 (0.90- 1.06)	0.987 (0.90-1.07)
*Equivalised income*			
*2nd*	0.898 (0.72-1.11)	0.876 (0.67-1.13)	0.881 (0.66-1.17)
*3rd*	0.816 (0.64-1.02)	0.946 (0.72- 1.23)	1.07 (0.80-1.43)
*4th*	0.768^b^ (0.60-0.98)	1.00 (0.75-1.33)	1.01 (0.74-1.38)
*Highest*	0.753 ^b^ (0.57-0.97)	0.870 (0.64- 1.18)	0.977 (0.69- 1.38)
*Urban*	0.908 (0.78- 1.03)	0.831^b^ (0.70- 0.97)	0.882 (0.74-1.04)
Observations	7587	6417	6115

Notes: The table presents estimated odds ratios from three separate binary logit models (1) “*MisclassifyW2”*using wave 2 of the GUI Infant Cohort, (2)“MisclassifyW3” and (3) MispredictW3 using wave 3 of the GUI Infant Cohort.. ^a^ denotes statistically significant at 1%, ^b^ denotes statistically significant at 5%, and ^c^ denotes statistically significant at 10%. Confidence Intervals in parathensis. Analysis is weighted to be nationally representative

The multivariate logistic regression analysis revealed a number of significant predictors of misclassification of child BMI status. In both waves of data, when the children were three years and five years old respectively, girls had increased odds of being inaccurately identified by mothers as being of normal weight, (OR =1.24, 95% confidence interval: 1.09-1.42) and (OR=1.36, 95% confidence interval: 1.17- 1. 59) compared to boys. Similarly children with a higher birth weight had increased odds of being misclassified by their mothers for both waves. Higher maternal BMI was associated with increased odds of mothers failing to identify their child as either overweight or obese (OR =1.50, 95% confidence interval: 1.28- 1.77) and OR =1.72, 95% confidence interval: 1.43- 2.06) respectively.

Likewise, in both waves, misclassification of child’s weight status was significantly associated with mothers who were working (albeit only at the 10% level for wave 3). In wave 2, the older the gestation age of the child in weeks, the lower the odds of mothers misclassifying their child’s weight status (OR: 0.92, 95% confidence interval: 0.88-0.96). Families who had private health insurance and households with higher incomes had lower odds of misclassifying their child’s weight status (OR: 0.78, 95% confidence interval: 0.65-0.92) and OR: 0.24, 95% confidence interval: 0.57-0.97respectively). Gestation age, private health insurance and household income were not significant predictors of misclassification for children when they are five years old. Families who lived in an urban area and mothers who had a postgraduate education were significantly less likely to misclassify their child at five years old. Location and maternal education were not significant for children when they were three years old.

Model 3 indicated that mothers who misclassify their child weight at three years old had much higher odds (OR: 7.26, 95% confidence interval: 0.13-8.59) of doing so again when the child is five years old. Visits to healthcare professionals by children and their mothers did not have any significant association with mothers’ ability to correctly identify their child’s weight status at either three or five years old. Other non-significant variables across both waves included self-reported health (both child and mother), maternal age, marital status, whether or not the child was breast fed, whether or not the child had siblings, also medical card status and also the number of visits the mother and child had taken to the GP, paediatric or consultant visits, public health nurse visits, practice nurse visits, and finally the number of overnight stays in hospital.

## Discussion

The purpose of this study was to investigate if mothers’ accurately identify their child’s overweight or obesity status at three years old and five years old, and also to examine if a failure to recognize overweight/obesity at three years old was associated with doing so again at five years old. The findings indicated that a significant proportion of mothers misclassified their child’s weight status in which they failed to accurately identify their child as overweight or obese. Findings also showed that, compared to when the child was three years old, there was a slight decrease in the percentage of mothers who misclassified their child’s weight when the child was five years old. Furthermore, mothers who inaccurately identified their child weight status at three years old were more likely to do so again at five years old.

The failure to identify overweight or obesity may be particularly evident for three to five year olds because the prevailing health message to mothers of young children is to encourage growth and keep pace with centile charts [31]. In line with the theory of cognitive dissonance, which proposes that people may try to reduce uncomfortable beliefs by rationalising them, it is possible that parents may choose to believe that their child will simply “grow out of it” and that their weight is not problematic and does not impact on child outcomes [27]. The issue of identification may be further clouded by the increased prevalence of overweight in children defining a “new” heavier norm against which parents compare their children [33]. Societal normalization of overweight is defined as a shift in the acceptability of overweight as normal [4, 7, 25]. In other words, mothers may have a higher threshold for defining what overweight means. Conversely, it may also be the case that parents do not wish to verbally label their child as overweight due to the negative connotations of the word ‘overweight’ [35, 38].

In the public health context, appropriately addressing maternal weight perceptions at different ages is vital. Therefore, determining if parental weight misclassification persists as a child gets older is important. Notably, this study found that mothers who misclassified their child’s weight at three years old are significantly more likely to do so when the child is five years old. While this may not be an entirely surprising finding, the implications of this suggest that educational interventions to inform mothers of healthy weight range during the early years might also lead to more accurate weight perceptions as the child gets older [42]. Furthermore, although health professionals are often the preferred source of child weight-related information [20], the finding in this study of a lack of any association between health care professionals and accurate maternal perceptions suggests that there are missed opportunities for healthcare professionals to improve knowledge exchange and early interventions to assist parents to recognize and support healthy weights for their children.

Positive parental changes occurs when a physical marker is visible, such as a diagnosis of childhood overweight or perceiving the child’s weight as a health problem [34]. One such example is the ‘Map Me’ intervention study which aimed to develop a visual method to improve parents’ ability to correctly assess their child’s weight status [23]. This study indicated that certain child, mother and household characteristics influence accurate perceptions of child weight status. Childhood obesity is disproportionately characteristic of low-income families. Developing tailored intervention programmes for the formative preschool cohort could prove to be a fruitful strategy to change attitudes and promote awareness of obesity in children within lower socioeconomic groups. Utilizing specific channels for health-related communications increases the likelihood that key messages will reach families who stand to benefit most from the information, and also improves the efficiency with which public funds for health communications are spent.

The majority of studies that have examined parental weight perception in preschool ages used self-reported BMI data [4, 7, 17]. Self-reported measures of weight and height can be under-reported and lead to bias [21]. Moreover, some studies used only partially measured data (obtaining the remainder from secondary sources such as medical records) [8]. Furthermore, some of these studies exclude important moderators that may influence parental estimates of child weight. For example, the association of many potentially important variables (e.g., child and parent ethnicity, parental gender, parental education, socioeconomic status) were not included in a meta-analyses that indicated that more than half of parents underestimated their children’s overweight/obese status [28]. Small sample size (*n*=281) [19] (*n*=120), [16] (n=230) and non-representative sample [6] are also some potential limitations of existing studies examining maternal perceptions of their child’s weight at preschool ages.

Strengths of this study include the use of a rich, nationally representative longitudinal survey of children living in Ireland, unlike other studies that use non-representative and often smaller sample sizes ([6, 26], [3, 13]). Thus, these results are generalizable to other settings. In addition, objective weight and height measures are used in contrast to many studies that use subjective weight measures to determine parental weight perceptions [17]. A key feature of the data in the current study is that the interviewer also measured weight and height for the child’s parents. A unique aspect of this study is the examination of early life conditions such as gestation age which have not been examined within a preschool cohort. Also distinctive to this study is the examination of the role that healthcare access and healthcare professionals play regarding accurate identification of child weight.

There are also some limitations to report. Firstly, given the analysis employed, results cannot be interpreted as causal relationships. Also, considering the combined manner in which the maternal perception question was asked, it was not possible to explore whether misclassification was different for overweight and obese children. Other limitations relate to BMI, which is criticised as an imperfect measure of adiposity, yet it is one of the best measures available for large scale use [22]. Also the children who were either overweight or obese were combined to ‘overweight’ and ‘obesity’ categories. Finally, due to attrition between waves, direct comparisons between waves should be treated with caution. Despite these limitations, our findings add to the current evidence base by examining weight identification during the preschool years.

## Conclusions

This study shows that during early childhood, mothers often failed to accurately identify their child’s overweight and/or obesity status. A range of child, mother and household characteristics contributed to maternal weight misperception when children are three years old and five years old. Misclassification of child weight at three years old was associated with misclassification of child weight at five years old. This has implications for the early detection and treatment of overweight and obesity; mothers unable to accurately identify children at risk are unlikely to act to prevent further excess weight gain. This is problematic given that 25% of three year olds and 20% of five-year-old children are overweight or obese in Ireland. This study suggests that health professionals and public health campaigns may need to play a greater role in terms of helping mothers’ correctly identify their child’s weight status.

## Abbreviations

BMI: Body Mass Index
GP: General Practitioner
GUI: Growing Up in Ireland
ICE: Interdisciplinary Capacity Enhancement
OR: Odds Ratio

## Footnotes

1 As part of the GUI study, parents were visited at home where they were asked to complete a questionnaire relating to health and lifestyle including their perception of their child’s weight status. The following sections were included in the questionnaire: Household Composition; Child’s Habits and Routines; Child’s Physical Health and Development; Parental Health; Child’s Play and Activities; Child’s Functioning and Relationships; Childcare Arrangements; Parenting and Family; Sociodemographic and finally Neighbourhood/Community. A primary caregiver sensitive questionnaire was also administered which detailed topics such as adoption, marriage details, alcohol consumption and smoking.

2 Some families emigrated between waves and therefore were not included in the useable sample. The useable sample for wave 2 was 10726 and for wave 3 it was 10360. This led to response rates of 91% for wave 2 and 86.9% for wave 3. There were also 794 families who participated in waves 1 and 3 only.

3 The main variables for which there was missing observations in wave 2 include: child BMI (3% missing), mother’s BMI (6% missing) and household income (5%). In wave 3, missing data was mainly observed for child BMI (7%), mother’s BMI (6%) and household income (5%). Other variables had smaller percentages of missing observations.

4 These were excluded if the mother’s stated that they perceived that there child was underweight. Children were also excluded if their clinical BMI category also placed them in the underweight category.

5 For wave 2: 0.57% (N=50) and wave 3: 0.88% (N= 75)

6 It is important to note that mothers were unaware when providing their survey questionnaire responses that the objective measures would follow. However, at five years, because it is repeated, parents might know that this is happening.
